# EZH2 Silencing with RNA Interference Induces G_2_/M Arrest in Human Lung Cancer Cells *In Vitro*

**DOI:** 10.1155/2014/348728

**Published:** 2014-03-18

**Authors:** Hui Xia, Wen Zhang, Yingjie Li, Nannan Guo, Changhai Yu

**Affiliations:** Department of Thoracic-Cardio Surgery, First Affiliated Hospital of PLA General Hospital, Beijing 100048, China

## Abstract

Nonsmall-cell lung cancer has a high mortality rate and poor prognosis. In the present study, we silenced EZH2 and explored the consequent cell cycle changes. The expression of cell-cycle-related proteins, including p53, p21, Cdc2, and cyclin B1, was detected with western blotting, and the cell cycle distribution was determined with flow cytometry. Inhibition of EZH2 expression changed the cell cycle distribution, in particular inducing G_2_/M arrest. Expression of Cdc2 and cyclin B1 was significantly decreased in A549 and HTB-56 cells after EZH2-siRNA treatment. In addition, p53 expression was increased by 21% and 18%, and p21 expression was increased by 31% and 23%, in A549 and HTB-56 cells, respectively, in the presence of EZH2-siRNA. This study clearly demonstrates that modulation of EZH2 expression with siRNA affects the cell cycle and the expression levels of p53 and p21, thereby changing cyclin B1 and Cdc2 expression and inducing G_2_/M arrest. These results may explain the observed antitumor activity of EZH2 silencing. Such explorations of the molecular mechanism of EZH2 will help us develop novel approaches to the diagnosis, treatment, and prevention of nonsmall-cell lung cancer.

## 1. Introduction

Lung cancer, the leading cause of cancer-related mortality worldwide, has a high mortality rate and poor prognosis. This is especially true for nonsmall-cell lung cancer (NSCLC), which accounts for approximately 80% of all lung cancers and has a 5-year overall survival rate <15% [1, 2]. In addition, approximately 40% of all patients diagnosed with NSCLC have unresectable stage III disease or medically inoperable disease [3]. Traditional therapeutic strategies, such as surgery, chemotherapy, and even radiotherapy, are the main modalities for NSCLC patients, but high systemic toxicity and drug resistance result in poor survival in most cases. Thus, enhancing patient survival by developing new therapeutic methods is urgently needed.

Recently, significant advances in our understanding of the biology and molecular mechanisms of cancer have allowed the development of molecular-targeted agents for the treatment of NSCLC. Some novel molecular targets in lung cancer include epidermal growth factor receptor and EZH2 combined with chemotherapy or radiotherapy [4, 5]. EZH2 is one of the targets currently being evaluated for the treatment of lung cancer; it is the catalytically active component of the PRC2 complex. EZH2 contains a SET domain with intrinsic histone lysine methyltransferase activity and directly interacts with and regulates the activity of the DNA methyltransferases DNMT1, DNMT3a, and DNMT3b [6]. EZH2 is widely expressed in developing embryos, and its expression decreases upon tissue maturation and differentiation. Various studies have shown that abnormal expression of EZH2, a potential marker to distinguish aggressive from indolent or benign cancers, is involved in the tumorigenesis of several types of malignancies, including melanoma and prostate, breast, bladder, and endometrial cancers [7]. EZH2 provides proliferative advantages to eukaryotic cells by interacting with the key pathways that control cell growth arrest and differentiation. Such oncogenic action of EZH2 overexpression can induce anchorage-independent colony growth and promote invasion* in vitro*. Furthermore, downregulation of EZH2 expression decreases the proliferation of cancer cells* in vitro* [8, 9]. However, how EZH2 promotes cell proliferation and tumor progression, including cell cycle distribution, is largely unknown.

The successful use of small interfering RNA (siRNA) to downregulate gene expression in several model systems has led to an increasing number of attempts to explore the potential use of this methodology in clinical settings. Knockdown of EZH2 with siRNA inhibits breast cancer cell proliferation, and pharmacological inhibition of EZH2 results in apoptosis of breast cancer cells [10]. In our previous reports, we showed that EZH2 silencing with RNA interference (RNAi) enhances A549 and HTB-56 cell sensitivity to irradiation both* in vitro* and* in vivo* and that this effect depends on the EZH2 expression level [11]. Inhibition of EZH2 with siRNA modulates changes in the cell cycle, but the underlying mechanism is not clear. Additionally, few reports have been published on the signaling pathway influencing the cell cycle after EZH2 silencing with siRNA in lung cancer cell lines. Exploration of the molecular mechanism of the antitumor efficacy of EZH2 siRNA will help us develop novel approaches for the diagnosis, treatment, and prevention of NSCLC.

The tumor suppressor protein p53 is a transcription factor that functions as a cellular gatekeeper and its mutation is often associated with the development of cancers. The p21 gene can be activated by p53 and induces cell cycle arrest owing to inhibition of kinase activity [12]. The expression of p53 and p21 proteins is crucial to cell cycle control, and tumor suppressor genes including these can modulate cell proliferation by modulating cell cycle progression [13]. Clinical evidence has shown that EZH2 expression levels are correlated with higher levels of p53 expression in gastric cancer, suggesting that their association should be further studied as possible predictive factors in gastric malignancies [14, 15]. Whether a relationship exists between EZH2 and the p53 pathway in lung cancer development is not clear. Thus, we hypothesized that modulation of EZH2 will affect the cell cycle via the p53 and p21 pathways.

In the present study, we silenced EZH2, which influenced the cell cycle and resulted in enhancement of therapeutic efficacy. We also explored the underlying mechanism and related pathway that modulates the cell cycle. Our results will provide direct theoretical evidence for application of EZH2-siRNA in the future clinical treatment of NSCLC patients.

## 2. Materials and Methods

### 2.1. Cell Lines and Culture

The human lung adenocarcinoma A549 and HTB-56 cell lines were obtained from the American Type Culture Collection (ATCC, Manassas, VA, USA) and were maintained in Dulbecco's modified Eagle's medium (DMEM, Gibco, Carlsbad, CA, USA) supplemented with 10% heat-inactivated fetal bovine serum and 1% penicillin/streptomycin at 37°C in a humidified incubator with 95% air and 5% CO_2_.

### 2.2. siRNA Transfection

A549 and HTB-56 cells were plated in six-well plates at a density of 2 × 10^5^ cells per well and grown overnight until they were 50–80% confluent to obtain maximum transfection efficiency. Cells were transfected with validated siRNA for EZH2 or a negative control vector (Qiagen, Lafayette, CO, USA) at a concentration of 100 nM using Lipofectamine 2000 transfection reagent (Invitrogen, Carlsbad, CA, USA). At 6 h after transfection, the medium was replaced with standard culture medium. The EZH2 gene targeting sequence was as follows: EZH2 sense, TTCATGCAACACCC AACACT; EZH2 antisense GAGAGCAGCAGCAAACTCCT.

After EZH2-siRNA transfection for 48 or 72 h, siRNA transfection efficiency was evaluated with green fluorescence analysis using a fluorescence microscope (OLYMPUS) and with western blotting to determine EZH2 expression. Briefly, A549 and HTB-56 cells were harvested and lysed in buffer containing 20 mM Tris-HCl (pH 7.5), 1% (w/v) Triton X-100, 150 mM NaCl, 1 mM EDTA, 1 mM *β*-glycerophosphate, 2.5 mM sodium pyrophosphate, 1 mM Na_3_VO_4_, and 1 *μ*g/mL leupeptin. Total cell protein extracts were obtained after centrifugation at 12,000 ×g for 30 min at 4°C. The protein concentrations of cell lysates were determined using a BCA protein assay kit (Pierce, Rockford, IL, USA). A standard western blotting protocol was used including SDS-PAGE separation and transfer to a polyvinylidene difluoride membrane. Each membrane was incubated with a primary antibody against EZH2 (1 : 1000, Cell Signaling Technology, Beverly, MA, USA) and anti-*β*-Actin. Each membrane was incubated with horseradish peroxidase-conjugated secondary antibody, and signal was visualized with ECL reagents.

### 2.3. Cell Cycle Analysis after EZH2 Silencing with RNAi

To test the influence of RNAi-mediated EZH2 silencing on the cell cycle, the distribution of cells in the different phases of the cell cycle was determined with flow cytometry and analyzed with CellQuest software version 3.3 (Becton Dickinson, San Jose, CA, USA). Briefly, after EZH2-siRNA transfection, the cells were harvested with trypsinization (1.125% w/v trypsin) and fixed with 70% ethanol (v/v) overnight at 4°C. The cells were then incubated with 100 *μ*g/mL RNase A and 50 mg/mL propidium iodide at room temperature for 30 min before cell cycle analysis. To further test the effect of EZH2 on G_2_/M arrest, cell cycles were synchronized at G_2_/M phase using nocodazole and then release them to detect the cell distribution changes [16, 17].

To determine whether the EZH2 expression level was indeed associated with the cell-cycle-related proteins cell division cycle 2 (Cdc2) and cyclin B1, expression of these proteins was evaluated using western blotting of A549 and HTB-56 cells after transfection with EZH2-siRNA. To further delineate the underlying mechanism of EZH2 silencing on modulation of the cell cycle, the expression of p53 and p21, which upregulate Cdc2 and cyclin B1, was analyzed with western blotting.

In order to prove EZH2 regulates cdc2 and cyclinB1 through p53 pathway, A549 and HTB-56 cells were transfected with control siRNA or p53-siRNA at a final concentration of 100 nM for 72 hours using Lipofectamine 2000 transfection reagent. Furthermore, the expression level of p53, p21, cdc2, and cycling B1 was analyzed through western blotting. A specific inhibitor of p53 (pifithrin-*α*, Tocris) or p21 (adaphostin, Tocris) was used for further investigation of the cell cycle and related protein changes.

## 3. Results

### 3.1. Evaluation of siRNA Transfection Efficiency

**Figure 1:**
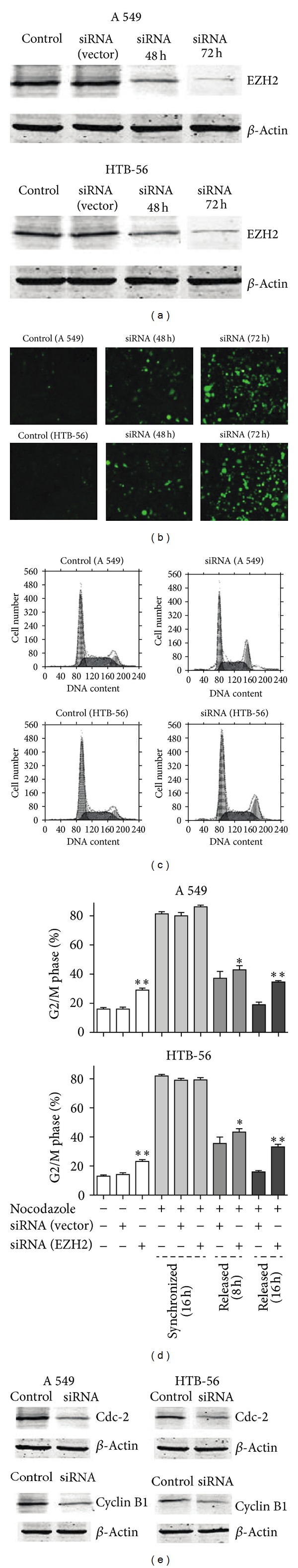
Influence of EZH2-siRNA on the cell cycle. (a) EZH2 protein expression was analyzed with western blotting after transfection with EZH2-siRNA for 48 or 72 h. (b) The transfection efficiency of EZH2-siRNA was observed after 48 or 72 h. (c) The changes in the cell cycle were observed with FACS analysis after transfection with EZH2-siRNA for 72 h. (d) The effect of EZH2-siRNA after synchronizing the cell cycles at G_2_/M phase using nocodazole. (e) After treatment with EZH2-siRNA, the expression of cell-cycle-related proteins, including Cdc2 and cyclin B1, was detected using western blotting. Each experiment was performed in triplicate.

Using western blotting, the expression of EZH2 in A549 and HTB-56 cells was evaluated after siRNA transfection. The results showed significant downregulation of EZH2 expression in a time-dependent manner. Compared with the control group, EZH2 expression decreased by 58% and 51% after 48 h and 87% and 75% after 72 h in A549 and HTB-56 cells, respectively (Figure 1(a)). These results were consistent with the result of the green fluorescence detection. Green fluorescence was greatly increased after 72 h of siRNA transfection compared to 48 h in both A549 and HTB-56 cells (Figure 1(b)). Thus, higher efficiency of EZH2-siRNA was obtained after 72 h of siRNA transfection, and we conducted transfections for 72 h in subsequent experiments.

### 3.2. Influence of EZH2-siRNA on Cell-Cycle-Related Proteins

The cell cycle distribution of A549 and HTB-56 cells was investigated with fluorescence-activated cell sorting (FACS) in the presence of control siRNA or EZH2-siRNA (Figure 1(c)). After 72 h of siRNA transfection, the S-phase and G_2_/M phase fractions of A549 and HTB-56 cells were clearly altered. The proportion of cells in G_2_/M increased by 13% and 9% after EZH2-siRNA treatment in A549 and HTB-56 cells, respectively. This result which was similar to our previous report [11] demonstrated that cell cycle progression was delayed or arrested in G_2_/M after knockdown of EZH2 with siRNA in A549 and HTB-56 cells.

A549 and HTB-56 cells were treated with 50 ng/mL nocodazole for 16 h to synchronize the cell cycles at G_2_/M phase. After that, cells were washed three times with phosphate-buffered saline and released into fresh medium for 8 h or 16 h, respectively. Flow cytometry was also used to evaluate the cell cycle distribution in the presence or absence of EZH2-siRNA. The results showed that cells were synchronized in G_2_/M phase by nocodazole treatment, and after following release from the nocodazole arrest, EZH2-siRNA shows the obvious effects on the G_2_/M arrest both in A549 and HTB-56 cells (Figure 1(d)). So far, the underlying mechanism of the above process has not been reported. Therefore, we next tested the possible signaling pathways that could influence such cell cycle changes after EZH2 silencing with RNAi.

Firstly, expression levels of Cdc2 and cyclin B1, which are directly related to changes in G_2_/M [18], were examined after EZH2-siRNA treatment of A549 and HTB-56 cells. After transfection with EZH2-siRNA, Cdc2 expression decreased by 21% and 14% in A549 and HTB-56 cells, respectively. And the inhibition on Cdc2 expression was clearly more effective in A549 cells than in HTB-56 cells. Furthermore, expression of cyclin B1 was also decreased by 14% and 12% after transfection of A549 and HTB-56 cells, respectively, but no significant difference was found in these two cell lines (Figure 1(e)). These results provided direct evidence that inhibition of EZH2 expression changes the cell cycle distribution of A549 and HTB-56 cells and in particular results in G_2_/M arrest, possibly by modulating the expression of Cdc2 and cyclin B1.

### 3.3. Influence of EZH2-siRNA on p53 and p21 Levels

**Figure 2:**
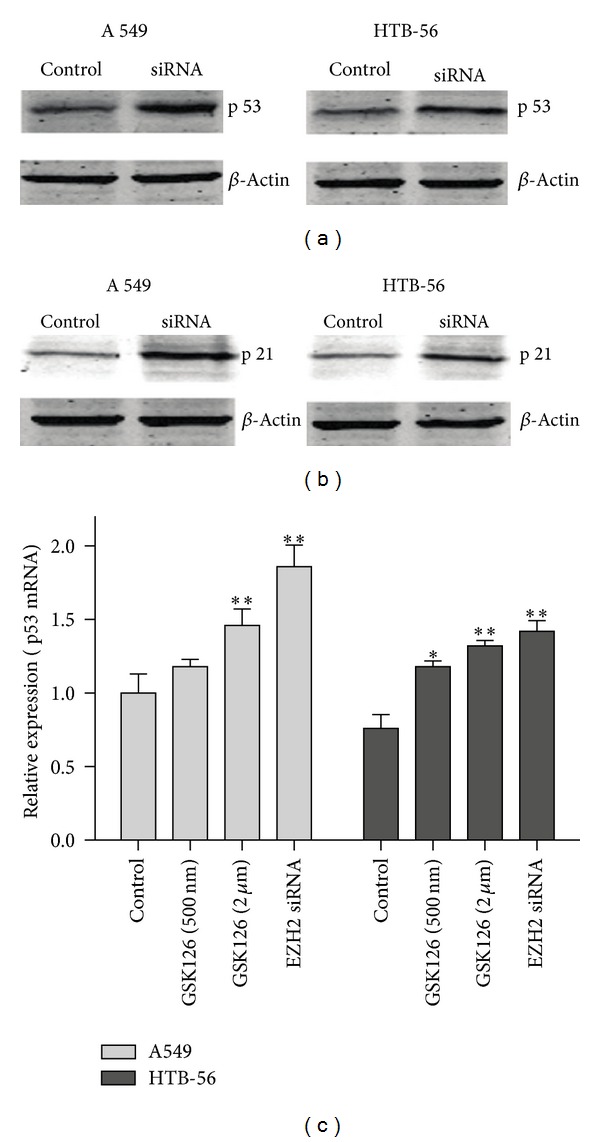
Effect of EZH2-siRNA on p53 and p21 protein expression. p53 expression (a) and p21 expression (b) were strongly increased in both A549 and HTB-56 cells after transfection with EZH2-siRNA. (c) Relative expression of p53 mRNA was detected in the presence of GSK 126 or EZH2-siRNA. Each experiment was performed in triplicate.

To examine the modulation of the Cdc2 and cyclin B1 pathway, we tested the changes in the levels of p53 and p21, which upregulate Cdc2 and cyclin B1 [19, 20], after EZH2-siRNA treatment of A549 and HTB-56 cells. Western blotting showed clear enhancement of p53 expression (21% and 18%) in A549 and HTB-56 cells, respectively, after transfection with EZH2-siRNA. In addition, the expression of p21 was also increased by 31% and 23% in A549 and HTB-56 cells, respectively, following EZH2-siRNA transfection (Figure 2). The enhancement of p21 expression was higher in A549 cells than in HTB-56 cells, but no clear difference was observed in p53 expression between the two cell lines. In addition, RT-PCR data also demonstrated that knockdown of EZH2 upregulates the p53 mRNA expression both in A549 (*P* < 0.01) and HTB-56 cells (*P* < 0.01) (Figure 2(c)). GSK126, which is a specific small molecular inhibitor for the catalytic SET domain of EZH2 [21, 22], will be helpful to reveal the relation between EZH2 and p53. In our experiments, GSK126 (2 uM) can enhance the p53 mRNA expression 1.4-fold (*P* < 0.01) in A549 cells and 1.7-fold (*P* < 0.01) in HTB-56 cells (Figure 2(c)).

**Figure 3:**
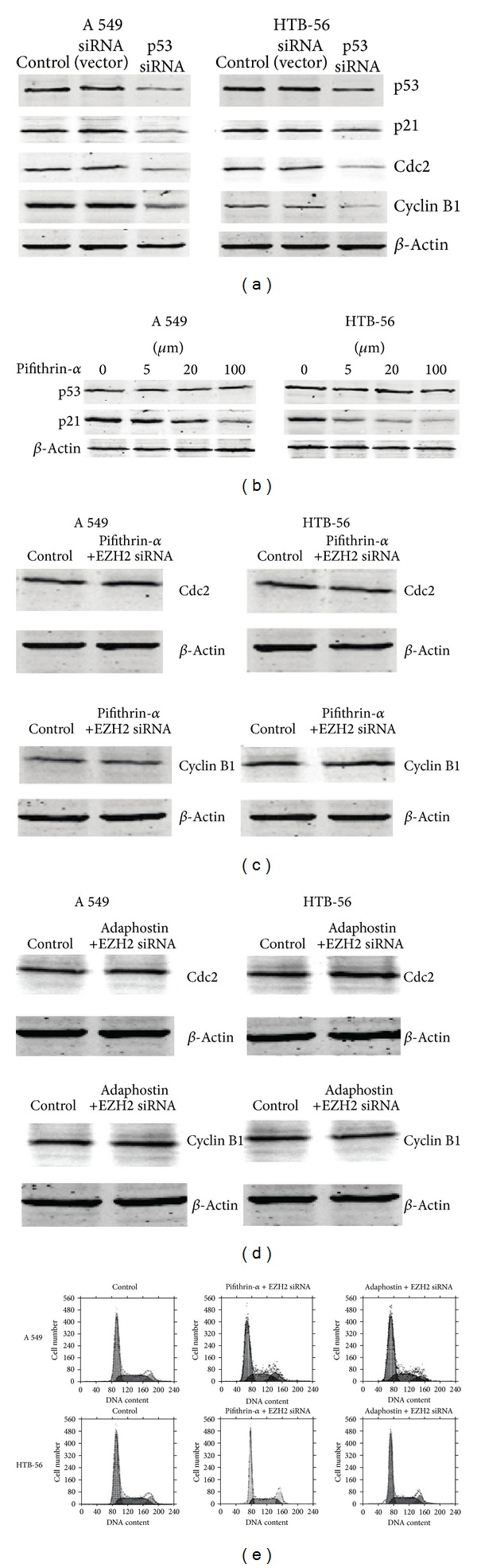
Effect of EZH2-siRNA on cell cycle progression via the p53 and p21 pathway. (a) The effects of p53-siRNA on the expression of downstream molecular were observed by western blotting. (b) p53 and p21 expression was detected after pifithrin-*α* application. (c) and (d) Cdc2 and cyclin B1 expression were detected with western blotting after incubation with a p53 inhibitor (pifithrin-*α*) (c) or p21 inhibitor (adaphostin) (d) in the presence of EZH2-siRNA. (e) The changes in cell cycle progression were observed with FACS analysis after incubation with the p53 or p21 inhibitor in the presence of EZH2-siRNA. Each experiment was performed in triplicate.

Based on these results, we hypothesized that the G_2_/M arrest and changes in the related proteins in human lung cancer cells are associated with p53 expression caused by EZH2-siRNA. Thus, we next used specific inhibitors of p53 and p21 following EZH2-siRNA transfection to clarify the mechanism of modulation of Cdc2 and cyclin B1 expression and cell cycle changes following EZH2 downregulation through the p53 and p21 pathways. It is evidenced that p53 depletion via siRNA transfection can effectively reduce the expression level of p53 and its downstream molecular, including p21, cdc2, and cyclin B1 (Figure 3(a)). In addition, pifithrin-*α* (PFT-*α*), a chemical inhibitor of p53 [23, 24], was also used to observe its downstream molecular expression through western blotting. After that, the expression of p21 was decreased by 43% and 65% in the presence of 20 uM pifithrin-*α*, in A549 and HTB-56 cells, respectively (Figure 3(b)). Further test was also performed in A549 and HTB-56 cells after pretreatment with 20 *μ*M pifithrin-*α* (p53 inhibitor), and the expression of Cdc2 and cyclin B1 was not changed in the presence of EZH2-siRNA. A similar result was observed after pretreatment with 10 *μ*M adaphostin (p21 inhibitor) (Figures 3(c) and 3(d)).

We also investigated the changes in the cell cycle with FACS after pretreatment with pifithrin-*α* or adaphostin following transfection with EZH2-siRNA. No significant changes were observed in the fractions of A549 or HTB-56 cells in the S-phase or G_2_/M phase (*P* > 0.05) (Figure 3(e)).

## 4. Discussion

Owing to limitations of traditional therapeutic treatments for NSCLC patients, such as toxicity and resistance to radiation and chemotherapy, development of more effective and less toxic treatments is urgently needed in current clinical lung cancer therapies. New modalities are continuously being sought via molecular biological, genetic, and immunological methods with the goal of enhancing the survival and quality of life of NSCLC patients. Some reports have been published about overexpression of EZH2 in several aggressive types of malignancies [25, 26], and the effect of EZH2 on cell cycle modulation was related to cyclin-dependent kinase (CDK) activity and retinoblastoma protein (pRB) in breast cancer and osteosarcoma [27, 28], but little information is available regarding the underlying mechanism of the effects of EZH2 on cell cycle modulation in lung cancer. Therefore, the purpose of the present work was to test the hypothesis that the influence of EZH2 on the cell cycle is modulated through p53- or p21-related proteins in lung cancer cell lines* in vitro*. Such results could lead to improved therapeutic efficacy.

In the present study, after transfection of EZH2-siRNA into A549 and HTB-56 cells, we report the following novel findings. (1) A clear influence on the cell cycle was observed in the presence of EZH2 silencing in these human lung cancer cell lines. (2) This modulation was related to decreases in cyclin B1 and Cdc2 expression. (3) Through its SET-domain regulated H3K27me3 activity, the effect of EZH2 on the cell cycle was related to the expression level of p53 and p21 and may underlie the mechanism of the antitumor activity of EZH2 silencing. Thus, our data demonstrate that inhibiting EZH2 may modulate the cell cycle through p53 and p21 signaling pathways.

We observed effective transfection of EZH2-siRNA into A549 and HTB-56 cells in our current experiment, which provided the basis for further research. Such inhibition of EZH2 delayed cell cycle progression and induced S and G_2_/M phase arrest. Modulation of DNA repair following the use of DNA-damaging agents will be influenced owing to the above changes in the cell cycle [29]. Cell-cycle-related proteins and their associated pathways were investigated to clarify the underlying mechanism. Some cell-cycle-related biomarkers, such as Cdc2 and cyclin B1, can regulate changes in the S phase and the G_2_/M transition and play an important role during cell cycle progression [30, 31]. Thus, the expression levels of Cdc2 and cyclin B1 in A549 and HTB-56 cells were evaluated using western blotting after EZH2 silencing with RNAi. In both cell lines, the expression of Cdc2 and cyclin B1 was strongly decreased 72 h after transfection. Thus, our direct evidence showed that EZH2-siRNA treatment modulated the distribution of cells in various stages of the cell cycle, possibly by affecting Cdc2 and cyclin B1. Interestingly, the inhibition of Cdc2 was stronger in A549 cells, but no clear difference was found between these two cell lines for cyclin B1 expression. These data are consistent with our previous report in which the expression levels of EZH2 in A549 and HTB-56 cells, which induces different therapeutic efficacies in terms of cell cycle progression, were different after siRNA treatment; Cdc2 expression was also different between the two cell lines. Such divergence in Cdc2 expression also suggests that the therapeutic efficacy of EZH2 silencing in clinical therapy may also be dependent on the underlying specific lung cancer pathology.

To the best of our knowledge, downregulation of EZH2 expression and its role in cell cycle arrest and related signaling pathways have not been completely explained in human lung cancer. Therefore, our study explored the underlying mechanism and provides a clear link and direct evidence between EZH2 expression and induction of cell cycle arrest in human lung cancer. Cell cycle progression is determined by checkpoints between early and late G1 and between the S and G_2_/M [32]. The tumor suppressors, p53 and p21, are involved in cell cycle progression [33]. p21 is the most important protein involved in cell cycle arrest at both the G_1_ and G_2_/M checkpoints. An important function of p53 is to bind to p53-specific DNA consensus sequence genes and increase the synthesis of p21. The G_2_/M checkpoints, which mediate growth arrest, can be modulated by p53, and the induction of p21 causes subsequent arrest at G_1_/G_0_ or G_2_/M by binding of the cyclin-CDK complex [34, 35]. We hypothesized that the modulation of Cdc2 and cyclin B1 expression after EZH2 inhibition is related to the p53 and p21 pathways. To test this hypothesis, the effect of EZH2 silencing on p53 and p21 expression levels was observed, and specific inhibitors of p53 and p21 were used in the presence of EZH2-siRNA. Strong enhancement of p53 and p21 expression was found after transfection with EZH2-siRNA in both A549 and HTB-56 cells, demonstrating that the antitumor effect and cell cycle arrest following EZH2 inhibition could be induced through the p53 and p21 pathways. More effective modulation of p21 expression was more observed in HTB-56 cells than in A549 cells, and this may partly reflect the different action of the antitumor effect after EZH2 inhibition.

Furthermore, to test whether EZH2 inhibits p53 through its SET-domain regulated H3K27me3 activity, GSK126, a potent, highly selective inhibitor of EZH2 methyltransferase activity, was used to observe the effect of EZH2 inhibition on the p53 mRNA expression in A549 and HTB-56 cells. Similar results were obtained compared with EZH2-siRNA application, and such specific inhibitor of EZH2 can also enhance p53 mRNA expression in the above two cell lines, which means EZH2 inhibits p53 through its SET-domain regulated H3K27me3 activity.

The application of specific inhibitors of p53 and p21 provided more details about this modulation. Firstly, the p53-siRNA was used to test whether EZH2 regulates Cdc2 and cyclinB1 through p53 pathway in A549 and HTB-56 cells. After p53-siRNA transfection, the expression level of its downstream molecular, including p21, cdc2, and cycling B1, was decreased obviously both in A549 and HTB-56 cells. Pifithrin-*α* (PFT-*α*), a chemical inhibitor of p53, was shown to specifically block transcriptional activity of the tumor suppressor p53 and protect against a variety of genotoxic agents. In our experiments, after treating the A549 and HTB-56 cells with pifithrin-*α*, the p21 expression was decreased both in A549 and HTB-56 cells. And p21 which is a gene target of p53 can also induce the following effect on Cdc2 and cyclinB1 expression. The above results provide the direct evidence that inhibition of p53 expression will play a key role in the modulation between EZH2 and cell-cycle-related protein. The western blotting and FACS results also showed that when the p53 and p21 pathway was blocked by specific inhibitors, EZH2 inhibition did not influence the cell cycle, Cdc2 or cyclin B1 expression, or arrest at G_2_/M. This result is consistent with a previous report showing that upregulation of p53 expression blocks cancer cell proliferation and changes the cell cycle [36].

In summary, we have shown for the first time that inhibition of EZH2 increased p53 expression in human lung cancer cells. Such upregulation of p53 likely changed the expression level of p21 and modulated the levels of cell-cycle-related biomarkers including Cdc2 and cyclin B1, thereby inducing G_2_/M arrest. In our current study, a basic mechanism for the anticancer effect of EZH2 silencing with RNAi was provided in human lung cancer cells. This information will be helpful for developing novel therapeutic applications of EZH2-targeted treatment of NSCLC patients.
